# Impact of Lysine-Mediated C-Terminal Dimerization of Anoplin on Killing Kinetics and Antibiotic Interaction Profiles

**DOI:** 10.3390/microorganisms14081681

**Published:** 2026-07-31

**Authors:** Stéfanne Rodrigues Rezende Ferreira, Eli Júnior Pereira Rodrigues, Guilherme Sastre de Souza, Ludimila Paula Vaz Cardoso, Eduardo Maffud Cilli, Esteban Nicolas Lorenzon, Hanstter Hallison Alves Rezende

**Affiliations:** 1 Institute of Health Sciences, Federal University of Jataí, Jataí 75801-615, GO, Brazil; stefanne2014@gmail.com (S.R.R.F.); eli.rodrigues@discente.ufj.edu.br (E.J.P.R.); guisastres@gmail.com (G.S.d.S.); ludimilacardoso@ufj.edu.br (L.P.V.C.); esteban@ufj.edu.br (E.N.L.); 2 Department of Biochemistry and Organic Chemistry, Institute of Chemistry, São Paulo State University (UNESP), Araraquara 14800-060, SP, Brazil; cilli@iq.unesp.br

**Keywords:** antimicrobial peptides, bacterial resistance, dimerization, synergism, anoplin

## Abstract

Antimicrobial resistance (AMR) limits the efficacy of conventional antibiotics, particularly in infections caused by multidrug-resistant bacteria. anoplin, a short membrane-active antimicrobial peptide, represents a promising scaffold for structural optimization. This study evaluated the antimicrobial performance of anoplin and its C-terminal lysine-mediated dimer, (anoplin)_2_K, against clinically relevant Gram-positive and Gram-negative bacteria, including carbapenemase-producing and non-producing *Klebsiella pneumoniae* strains. Minimum inhibitory concentration (MIC), minimum bactericidal concentration (MBC), time–kill kinetics, checkerboard interaction assays, and hemolytic activity were determined. While MIC values showed moderate differences between the peptides, (anoplin)_2_K demonstrated enhanced bactericidal kinetics, achieving complete eradication of *Escherichia coli*, *Staphylococcus aureus*, and *Pseudomonas aeruginosa* within 60 min at 256 µM. Additive effects predominated in combination assays, with selective synergism observed for sulfamethoxazole–trimethoprim. Reduced susceptibility was observed for carbapenemase-producing *K. pneumoniae*. Although the dimeric analogue exhibited increased hemolytic activity at higher concentrations, the results indicate that C-terminal dimerization modulates killing dynamics and enhances antimicrobial performance. These findings support further optimization of anoplin-derived dimers as potential adjunct strategies against multidrug-resistant pathogens.

## 1. Introduction

Infections caused by multidrug-resistant (MDR) bacteria are a major concern for global health. The growing prevalence of antimicrobial resistance (AMR), combined with pharmacological limitations that hinder the penetration of antibiotics into tissues such as the central nervous system, the lungs, and the bones, significantly reduces therapeutic success [1,2]. Among clinically relevant Gram-negative pathogens, *Klebsiella pneumoniae* is a major cause of healthcare-associated and community-acquired infections, including pneumonia, bloodstream infections, and urinary tract infections. The spread of strains that produce extended-spectrum β-lactamases and are resistant to carbapenems has significantly limited therapeutic options. Of particular concern is the emerging convergence between antimicrobial resistance and hypervirulence, resulting from the acquisition of resistance determinants, including extended-spectrum β-lactamases and carbapenemases, by hypervirulent strains [3].

*Acinetobacter baumannii* is also one of the main factors contributing to the global burden of AMR, particularly in hospital and intensive care settings. Its clinical relevance is associated with its ability to persist on abiotic surfaces, colonize medical devices, form biofilms, and acquire multiple resistance determinants. Isolates resistant to carbapenems and extensively resistant to antimicrobials are especially difficult to treat due to their reduced susceptibility to various first-line and last-resort antimicrobial agents [4]. Taken together, these challenges highlight the urgent need for alternative antimicrobial strategies capable of overcoming both microbiological resistance and pharmacokinetic constraints.

Antimicrobial peptides (AMPs) have emerged as promising candidates due to their broad-spectrum activity, rapid bactericidal action, and lower likelihood of resistance development compared to conventional antibiotics [5,6]. Many AMPs exert their antimicrobial effects through membrane-targeting mechanisms, including membrane destabilization and pore formation, which differs fundamentally from the specific intracellular targets of classical antibiotics.

Peptides derived from venoms constitute an important subgroup of AMPs, particularly those isolated from Hymenoptera species, which have been increasingly investigated as potential therapeutic platforms due to their structural simplicity and potent membrane activity [7]. Among them, anoplin (GLLKRIKTLL-NH_2_), a linear amphipathic peptide composed of 10 amino acids isolated from the venom of *Anoplius samariensis*, is one of the smallest naturally occurring α-helical AMPs described to date [8]. Its cationic structure allows for electrostatic interactions with negatively charged bacterial membranes, resulting in membrane destabilization and cell lysis.

Despite its promising biological activity, anoplin has limitations that restrict its therapeutic application, including susceptibility to proteolytic degradation and potential cytotoxicity at higher concentrations [9]. To overcome these limitations, various structural modification strategies have been explored, including rational amino acid substitutions, lipidation, dendrimerization, and multimerization approaches aimed at improving antimicrobial potency and stability [10,11]. In particular, it has been demonstrated that lysine-based dendrimerization, combined with N-terminal lipidation, significantly modulates the antimicrobial activity and selectivity of anoplin derivatives [12].

Peptide dimerization has been described as a promising strategy for enhancing antimicrobial activity by increasing the local concentration of the peptide, improving membrane affinity, and modulating structural orientation [13]. In the case of anoplin, previous studies have demonstrated that the dimerization site can significantly influence biological activity, highlighting the importance of structural configuration in determining antimicrobial performance [14]. Furthermore, the rational optimization of short AMPs has shown that subtle structural modifications can markedly affect antimicrobial and antibiofilm activity profiles [11].

In this study, we evaluated the antibacterial activity of anoplin and its C-terminal lysine-mediated dimer, (anoplin)_2_K, against clinically relevant Gram-positive and Gram-negative bacteria, including *Staphylococcus aureus*, *Enterococcus faecalis*, *Escherichia coli*, *Pseudomonas aeruginosa* and carbapenemase-producing and non-producing strains of *K. pneumoniae*. The minimum inhibitory concentration (MIC), minimum bactericidal concentration (MBC), time–kill kinetics, hemolytic activity and combinatorial interactions with conventional antibiotics were determined to assess the microbiological impact of dimerization.

## 2. Materials and Method

### 2.1. Peptide Synthesis and Characterization

Anoplin and (anoplin)_2_K were manually obtained by solid-phase peptide synthesis (SPPS) using the standard Fmoc (9-fluorenylmethyloxycarbonyl) protocols on a Rink-amide resin. For the dimer, Fmoc-Lys(Fmoc)-OH was attached to the resin, and after α- and ε-Fmoc group deprotection with 20% piperidine/dimethylformamide (DMF), the two chains of (anoplin)_2_K were simultaneously elongated. For both anoplin and (anoplin)_2_K, the amino acids were coupled at twofold excess over the amino component in the resin, using diisopropylcarbodiimide (DIC)/N-hydroxybenzotriazole (HOBt) in 50% (*v*/*v*) DCM (methylene chloride)/DMF. After 2 h, the qualitative ninhydrin test was performed to estimate the completeness of the coupling reaction; the recoupling procedure was performed when the ninhydrin test was positive. Cleavage of the resin and removal of the side chain protecting groups for all the peptides were simultaneously performed with 95% TFA, 2.5% water and 2.5% TIS for 2 h. After this procedure, the crude peptides were precipitated with anhydrous ethyl ether, separated from soluble nonpeptide material by centrifugation, extracted into 0.045% (*v*/*v*) TFA/H_2_O (solvent A), and lyophilized. The crude peptides were dissolved in solvent A and purified by semi-preparative HPLC using a reverse-phase C18 column with a linear gradient 40–80% of solvent B (0.036% (*v*/*v*) TFA/acetonitrile) for 120 min. The flow rate was 5 mL/min. UV detection was carried out at 220 nm. The peptide homogeneity was checked by analytical HPLC, using solvents A and B with a linear gradient of 5–95% (*v*/*v*) of solvent B for 30 min, at a flow rate of 1.0 mL/min and UV detection at 220 nm. The identity of the peptide was confirmed by mass spectrometry in positive ion mode ESI on a Bruker model apparatus (Ettlingen, Germany).

### 2.2. Microbial Cells and Culture Conditions

All assays were performed at the Laboratory of Bacteriology and Mycology (LBM), Federal University of Jataí (UFJ), using commercial reference strains (Newprov^®^, Pinhais, Brazil): *Staphylococcus aureus* ATCC^®^ 25923 (ATCC 0023), *Enterococcus faecalis* ATCC^®^ 29212 (ATCC 0033), *Escherichia coli* ATCC^®^ 25922 (ATCC 0039), *Pseudomonas aeruginosa* ATCC^®^ 9027 (ATCC 0027), and *Klebsiella pneumoniae* ATCC^®^ BAA-1705 (KPC-positive) and ATCC^®^ BAA-1706 (KPC-negative). Strains were grown in Brain Heart Infusion broth (BHI, Oxoid^®^, Basingstoke, United Kingdom) at 35–37 °C for 18–24 h and streaked onto Mueller–Hinton agar (MHA, Himedia^®^, Thane, India) to confirm purity. Pure colonies were then used to prepare suspensions in sterile 0.9% saline, adjusted to the 0.5 McFarland standard (1 × 10^8^ CFU/mL), and diluted in Mueller–Hinton broth (MHB, Himedia^®^) to the concentrations required for each assay.

### 2.3. Antibacterial Activity

The minimum inhibitory concentration (MIC) was determined by broth microdilution in 96-well plates, using peptide concentrations ranging from 2 to 256 μM. Aliquots (50 μL) of bacterial suspension (1 × 10^6^ CFU/mL) were added to serial twofold dilutions of the peptides in Mueller–Hinton broth (Himedia^®^), with positive, negative, and known-drug controls. After incubation at 35–37 °C for 24 h, the MIC was defined as the lowest concentration showing no visible growth. The minimum bactericidal concentration (MBC) was determined from wells showing no growth at the MIC by plating onto Mueller–Hinton agar and incubating for 24 h; the MBC was considered the lowest concentration yielding no bacterial growth [15]. All assays were performed in triplicate.

### 2.4. Time–Kill Curve

Bactericidal activity over time was evaluated using the time-to-death assay [16]. Bacterial suspensions at 2 × 10^5^ CFU/mL were treated with the peptides at concentrations corresponding to their MBC values and incubated at 35–37 °C. Samples were collected at intervals ranging from 0 to 120 min, serially diluted in sterile saline, and plated on Mueller–Hinton agar for colony counting after incubation. Rifampicin and the diluent were used as positive and negative controls, respectively. Each assay was performed in triplicate using the same bacterial suspension, and the average bacterial count was used to plot the elimination time curves.

### 2.5. Synergism Assay

The checkerboard assay was used to evaluate the interaction between antimicrobial peptides (anoplin and (anoplin)_2_K) and conventional antibiotics [17]. Following BrCast and EUCAST recommendations [17,18] for determining the concentrations of the selected antimicrobials clindamycin, cefoxitin, and sulfamethoxazole–trimethoprim, the assay was performed in 96-well plates, with one agent serially diluted across the columns (horizontally) and the other across the rows (vertically), generating a matrix of combinations. Each well received 50 μL of bacterial suspension (5 × 10^5^ CFU/mL), for a final volume of 150 μL per well.

After incubation at 35–37 °C for 24 h, turbidity was assessed to determine the MIC of each agent alone and in combination. The fractional inhibitory concentration index (FICI) was calculated to classify the interactions as synergistic (≤0.5), additive (0.5–1), indifferent (1–2), or antagonistic (>2).

### 2.6. Hemolysis Assay

Human erythrocytes were separated from plasma by centrifugation, washed three times, and prepared as a 1% (*v*/*v*) suspension. Samples were incubated with increasing concentrations of the peptides, ranging from 2 to 512 μM, at 37 °C for 1 h. After centrifugation, 100 μL of the supernatant were transferred to 96-well microplates, and absorbance was measured at 405 nm. Complete hemolysis (100%) was defined using 1% (*v*/*v*) Triton X-100, and zero hemolysis (0%) using PBS. The concentration causing 50% lysis (HC_50_) was determined based on absorbance relative to the positive (Triton X-100) and negative (saline) controls. The assay was performed in triplicate.

### 2.7. Ethics Statement

All experimental procedures were previously approved by the Research Ethics Committee of the UFJ, under protocol No. 6.410.203/2023. Human blood collection followed the principles of Resolution No. 466/12 of the Brazilian National Health Council, with written informed consent obtained from all volunteers, ensuring anonymity and confidentiality.

## 3. Results

### 3.1. Peptide Synthesis and Characterization

Anoplin and (anoplin)_2_K were successfully synthesized via solid-phase peptide synthesis and purified by reverse-phase HPLC (Figure S1). Analytical HPLC confirmed the homogeneity of the peptides, and mass spectrometry verified the expected molecular masses of 1153.5 Da for anoplin and 2418.18 Da for (anoplin)_2_K (Figure S2). The purified peptides were subsequently used in antimicrobial assays.

### 3.2. Antibacterial Activity

The anoplin peptide exhibited high MIC values (256 µM) against the Gram-negative strains *Escherichia coli*, *Klebsiella pneumoniae* (KPC^+^ and KPC^−^), and *Pseudomonas aeruginosa*. In contrast, (anoplin)_2_K showed greater activity against *E. coli* and *P. aeruginosa* (128 µM), while maintaining a high MIC for *K. pneumoniae*. For the Gram-positive strains (*Staphylococcus aureus* and *Enterococcus faecalis*), both anoplin and (anoplin)_2_K displayed low efficacy, with MICs ≥ 256 µM (Table 1).

Similarly to the MIC results, the MBC values indicated higher effectiveness of (anoplin)_2_K against *E. coli* and *P. aeruginosa* (128 µM), whereas *K. pneumoniae* remained resistant. The Gram-positive strains showed MBC values above 256 µM for both peptides, indicating low bactericidal activity against these bacteria.

### 3.3. Time–Kill Curve

The bactericidal activity of anoplin and its dimeric analogue (anoplin)_2_K was assessed by monitoring bacterial viability, expressed as log_10_ CFU/mL, over 120 min. Test concentrations were based on previously determined MIC and MBC values.

**Figure 1:**
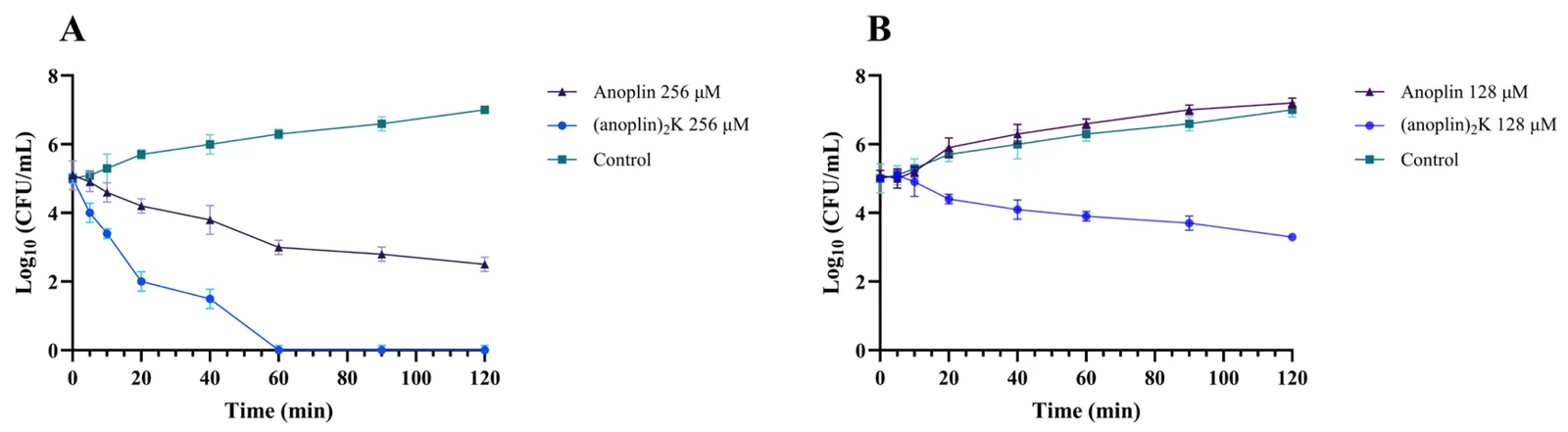
Time–kill curves for *Escherichia coli* treated with anoplin and (anoplin)_2_K at (**A**) 256 µM and (**B**) 128 µM.

For *E. coli*, the positive growth control showed continuous proliferation, reaching approximately 7 log_10_ CFU/mL by the end of the experiment. Treatment with anoplin at 256 µM promoted a marked reduction during the first 40 min, stabilizing at around 3 log_10_ CFU/mL up to 120 min. In contrast, (anoplin)_2_K completely eradicated the bacterial population within 60 min, demonstrating greater bactericidal efficacy at this concentration (Figure 1A). At 128 µM, anoplin failed to prevent bacterial growth, while (anoplin)_2_K reduced cell counts to approximately 3 log_10_ CFU/mL, indicating superior performance even at subinhibitory concentration (Figure 1B).

**Figure 2:**
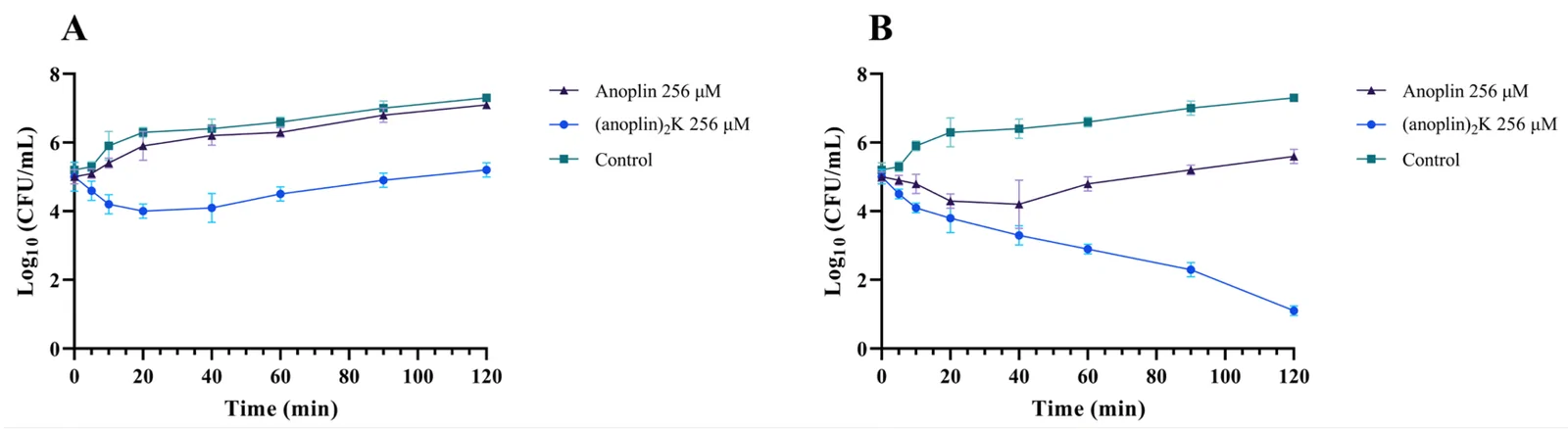
Time–kill curves for *Klebsiella pneumoniae* of KPC-positive (**A**) and KPC-negative (**B**).

For the KPC-positive *K. pneumoniae* strain (Figure 2A), both peptides caused an initial reduction in bacterial viability; however, only (anoplin)_2_K maintained partial suppression, with bacterial counts remaining at approximately 5 log_10_ CFU/mL at the end of the experiment. Against the KPC-negative strain (Figure 2B), anoplin at 256 µM caused only a slight initial reduction, followed by bacterial regrowth. In contrast, (anoplin)_2_K produced a more sustained reduction, reaching approximately 1 log_10_ CFU/mL at the end of the assay. These distinct kinetic profiles indicate strain-dependent differences in susceptibility, despite exposure to the same peptide concentration.

**Figure 3:**
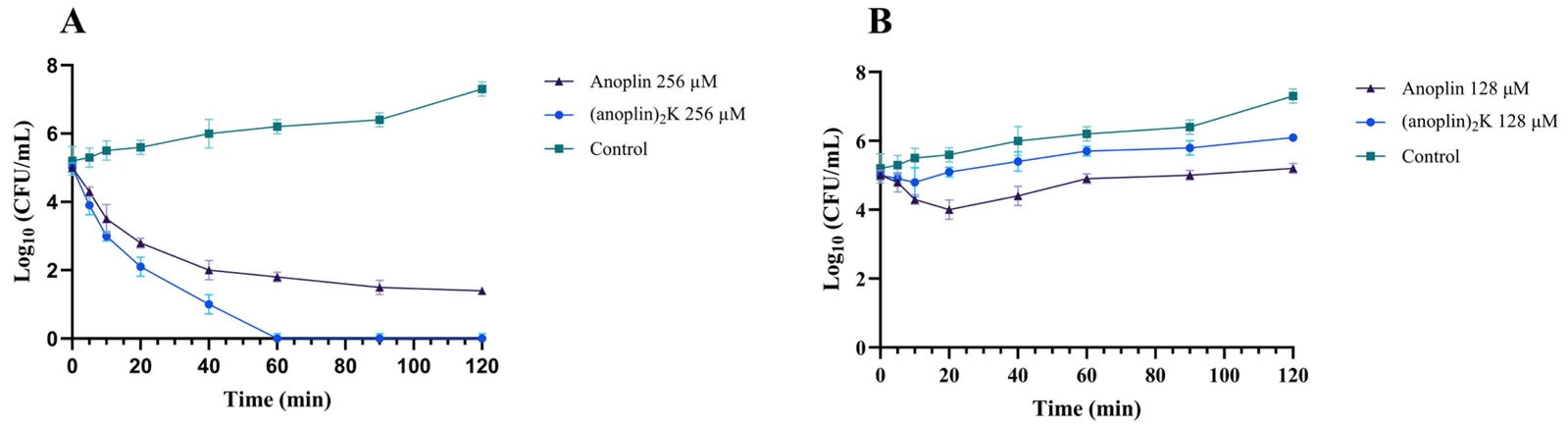
Time–kill curves for *Pseudomonas aeruginosa* treated with anoplin and (anoplin)_2_K at (**A**) 256 µM and (**B**) 128 µM.

Against *P. aeruginosa*, anoplin at 256 µM reduced viability to around 2 log_10_ CFU/mL, while (anoplin)_2_K achieved complete eradication within 60 min (Figure 3A). At 128 µM, both peptides had limited effects, although (anoplin)_2_K still outperformed the monomer (Figure 3B).

**Figure 4:**
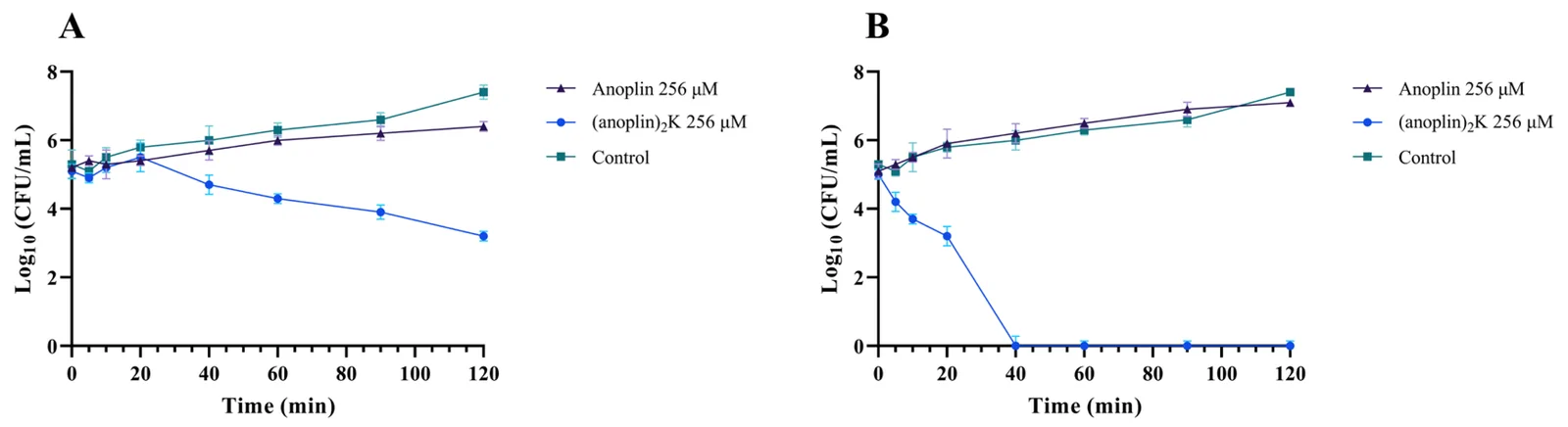
Bactericidal activity of the peptides against *Enterococcus faecalis* (**A**) and *Staphylococcus aureus* (**B**).

For Gram-positive strains, anoplin showed no relevant activity against *E. faecalis*, with continuous growth reaching 5.5 log_10_ CFU/mL, whereas (anoplin)_2_K induced sustained reduction to approximately 3 log_10_ CFU/mL by the end of the experiment (Figure 4A). Against *S. aureus*, anoplin maintained viability above 6 log_10_ CFU/mL, while (anoplin)_2_K exhibited marked bactericidal activity, with complete eradication observed within 40 min (Figure 4B).

### 3.4. Synergism Assay

(anoplin)_2_K showed predominantly additive effects when combined with antibiotics such as cefoxitin, sulfamethoxazole–trimethoprim, and clindamycin. A specific synergistic interaction was observed between (anoplin)_2_K and sulfamethoxazole–trimethoprim against *E. coli*. Conversely, some combinations displayed antagonism, such as the high-concentration association with *E. coli.* For *P. aeruginosa* and *E. faecalis*, the effects were either indifferent or absent (Table 2).

### 3.5. Hemolysis Assay

**Figure 5:**
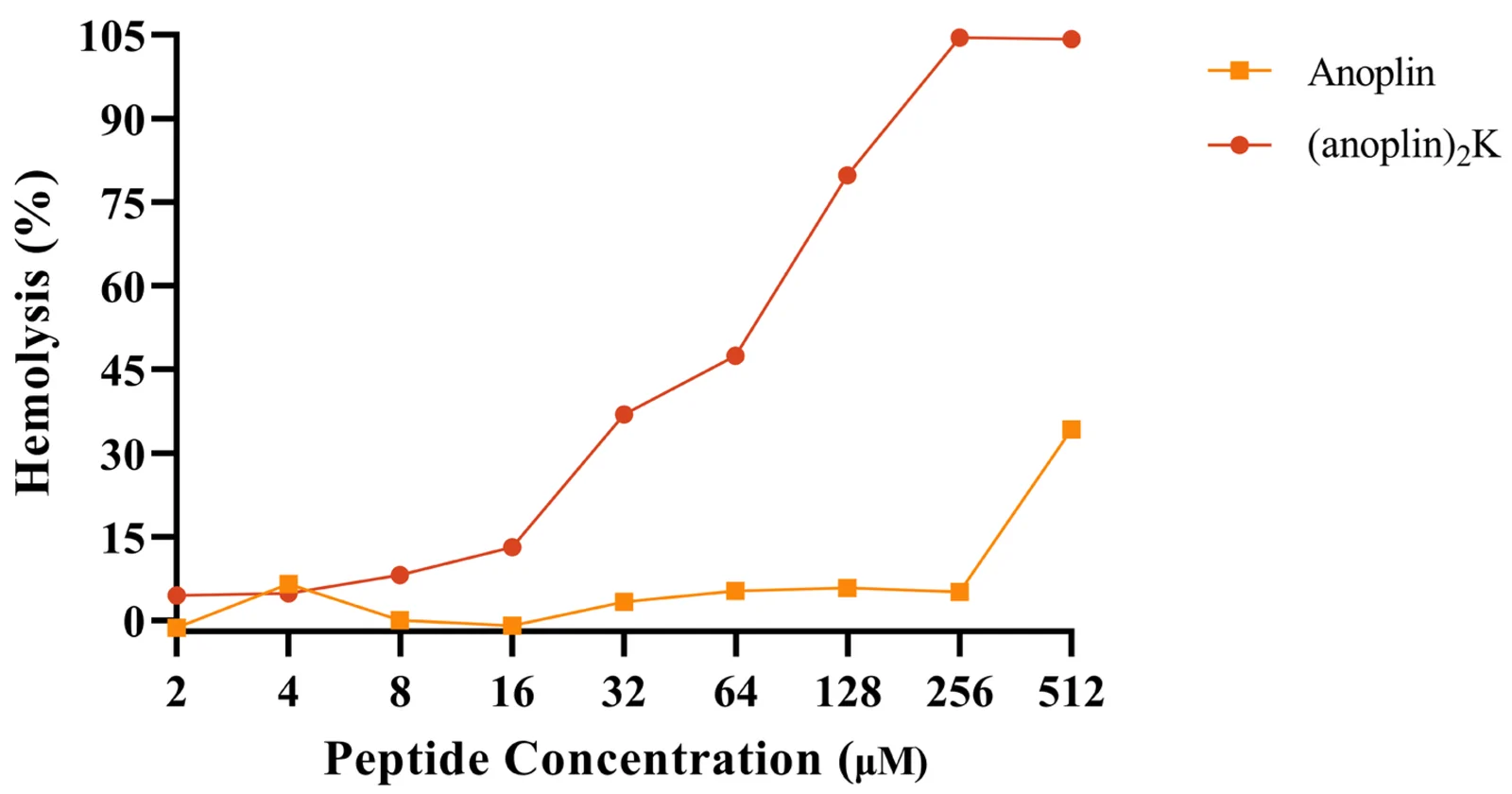
Hemolytic activity of anoplin and (anoplin)_2_K.

A 1% solution of human erythrocytes was used to determine the concentration causing 50% hemolysis (HC_50_) for the tested peptides. The HC_50_ value for anoplin was greater than 512 µM; 50% hemolysis was not reached within the tested concentration range. In contrast, (anoplin)_2_K exhibited an EC_50_ of approximately 64 µM, indicating a higher hemolytic potential. These results suggest that dimerization significantly increased the peptide’s interaction with erythrocyte membranes, thereby increasing its toxicity (Figure 5).

## 4. Discussion

In this study, we compared the antimicrobial performance of anoplin and its C-terminal lysine-mediated dimer, (anoplin)_2_K, against clinically relevant Gram-positive and Gram-negative pathogens, including carbapenemase-producing *K. pneumoniae*. The results demonstrate that dimerization markedly enhanced bactericidal kinetics, particularly against *E. coli*, *P. aeruginosa* and *S. aureus*. The rapid eradication observed at 256 µM suggests that increased local peptide density and improved membrane interaction contributed to accelerated membrane disruption. These findings are consistent with previous reports showing that structural modification and dimerization of AMPs enhance membrane interaction and permeability [19,20,21].

Previous work by Gou et al. demonstrated that lysine-based dendrimerization combined with N-terminal lipidation significantly enhanced the antimicrobial activity of anoplin derivatives, particularly through increased hydrophobic interactions and altered peptide topology [12]. In contrast to the dendrimeric and lipidated structures described in that study, the present work evaluates a simpler C-terminal lysine-mediated linear dimerization strategy. While Gou et al. [22] reported substantial reductions in MIC values following structural modification, our findings emphasize modulation of bactericidal kinetics rather than dramatic shifts in inhibitory concentration. This distinction suggests that different multimerization architectures may differentially influence potency, selectivity, and killing dynamics.

The different inactivation profiles observed between KPC-negative and KPC-positive *K. pneumoniae* strains, despite exposure to the same peptide concentration, likely reflect strain-dependent differences in cell envelope composition and resistance-associated phenotypes. Although KPC production alone is not expected to directly inactivate antimicrobial peptides, KPC-producing strains often exhibit additional adaptive mechanisms, including altered outer membrane permeability, changes in lipopolysaccharide and capsule composition, loss of porins, and increased efflux activity [23]. These characteristics may reduce the peptide’s access to the cytoplasmic membrane or limit membrane disruption. Thus, although (anoplin)_2_K produced a more sustained reduction in bacterial count against the KPC-negative strain, only partial suppression was observed against the KPC-positive strain. Therefore, the reduced and less sustained activity against the KPC-positive isolate may be associated with its broader resistance phenotype, rather than solely with carbapenemase production.

The moderate activity observed against *E. faecalis* and the limited activity of monomeric anoplin are in agreement with the literature indicating that simple linear peptides often exhibit reduced efficacy against Gram-positive cocci with thick peptidoglycan layers and limited membrane permeability [22,24,25]. In contrast, the strong activity of (anoplin)_2_K against *S. aureus*, including rapid eradication within 40 min, highlights its potential relevance in infections where this pathogen plays a major role, particularly in biofilm-associated medical device infections [26].

The time–kill kinetics confirm that (anoplin)_2_K exerts a rapid and sustained bactericidal effect. This rapid membrane-targeting action is characteristic of AMPs and differs fundamentally from conventional antibiotics that inhibit intracellular enzymatic pathways or protein synthesis [27]. Such a mechanism may reduce the likelihood of resistance development due to its non-specific membrane-disruptive nature.

Combination assays revealed predominantly additive effects, with limited synergism. According to Pankey and Sabath [28], additive interactions can still provide clinical benefit, especially when targeting resistant strains. For *S. aureus*, the combination of (anoplin)_2_K with clindamycin resulted in modest improvement, whereas certain combinations with sulfamethoxazole–trimethoprim showed synergism against *E. coli* and *K. pneumoniae*. In contrast, all combinations against *P. aeruginosa* were classified as indifferent, consistent with previous observations that membrane permeability barriers and intrinsic resistance mechanisms limit synergistic interactions in this species [29].

Hemolytic assessment demonstrated that (anoplin)_2_K exhibited increased erythrocyte lysis at 512 µM, whereas monomeric anoplin maintained a safer hemolytic profile, corroborating previous reports describing its low hemolytic potential under similar conditions [30]. The increased hemolysis observed for the dimer likely reflects enhanced hydrophobic interactions with mammalian membranes, highlighting the trade-off between potency and selectivity commonly observed in AMP engineering. Although the hemolytic activity may limit systemic application, alternative approaches such as topical use, coating of medical, or encapsulation and controlled release systems could mitigate toxicity while preserving antimicrobial efficacy [31]. In addition to the structural optimization of antimicrobial peptides, the growing problem of antimicrobial resistance highlights the need for complementary strategies capable of circumventing conventional resistance mechanisms and reducing bacterial pathogenicity.

One promising approach is the so-called “Trojan horse” strategy, in which antibiotics are conjugated to molecules that exploit bacterial nutrient uptake systems to facilitate targeted delivery [32]. Siderophore-antibiotic conjugates, for example, take advantage of bacteria’s need for iron and the active transport of iron-chelating compounds across the outer membrane. Once internalized, the antibiotic component can reach its periplasmic or intracellular target, thereby helping to overcome permeability barriers, porin loss, and efflux-mediated resistance [33].

A related antivirulence strategy involves targeting bacterial metallophores and their associated biosynthesis, export, and uptake systems. Metallophores mediate the acquisition of essential metals required for bacterial growth, metabolism, biofilm formation, quorum sensing, and the activity of various metalloproteins and metalloenzymes. Therefore, inhibiting metallophore synthesis, the uptake of extracellular metals, receptor-mediated uptake, or the intracellular utilization of metals can impair bacterial fitness and virulence. Experimental evidence indicates that interfering with metal acquisition can simultaneously reduce the activity of multiple metal-dependent enzymes and suppress virulence-associated phenotypes, reinforcing the idea that metallophore pathways are promising therapeutic targets [34,35].

Bacteriophage-based antimicrobials represent another potential alternative or adjunctive strategy against multidrug-resistant pathogens. Lytic bacteriophages selectively infect susceptible bacterial cells, replicate within them, and induce bacterial lysis, while generally sparing non-target members of the microbiota [36]. Phage cocktails, genetically modified phages, phage-derived lytic proteins, and combinations of phages with antibiotics can enhance antibacterial activity and help overcome the limited host range and the emergence of phage resistance. In particular, the synergy between phages and antibiotics can increase bacterial elimination, reduce the required antibiotic concentrations, and potentially restore antimicrobial susceptibility in resistant strains. However, clinical implementation still requires careful analysis of phage specificity, formulation, administration, immune clearance, bacterial resistance to phages, and standardized susceptibility testing [37].

Together with AMP engineering, these approaches illustrate how the future development of anti-infective agents could integrate direct bactericidal activity, targeted drug delivery, antivirulence mechanisms, and pathogen-specific biological agents to improve the treatment of infections caused by multidrug-resistant bacteria. Overall, these results indicate that C-terminal lysine-mediated dimerization enhances the bactericidal kinetics and antimicrobial activity of anoplin, although with reduced selectivity at higher concentrations. Rather than simply increasing potency, this modification appears to modulate microbiological behavior, particularly elimination kinetics, which supports the need for further structural optimization to improve the therapeutic index.

## 5. Conclusions

This study demonstrated that C-terminal lysine-mediated dimerization enhances the antimicrobial performance of anoplin, particularly by accelerating the bactericidal kinetics against selected Gram-positive and Gram-negative pathogens. Although the minimum inhibitory concentrations were not significantly reduced, the dimeric analog exhibited faster and more pronounced bacterial eradication, highlighting the impact of structural multimerization on microbiological behavior. However, the increase in hemolytic activity at higher concentrations indicates a trade-off between potency and selectivity, underscoring the need for further structural optimization. Overall, these findings suggest that controlled dimerization strategies may represent a viable approach to modulating the functional properties of antimicrobial peptides and supporting their development as adjuvant or alternative agents against multidrug-resistant infections.

## Figures and Tables

**Table 1:** Minimum inhibitory and bactericidal concentrations of anoplin and (anoplin)_2_K against different bacterial strains.

Bacteria	MIC (µM)		MBC (µM)	
	Anoplin	(anoplin)_2_K	Anoplin	(anoplin)_2_K
Gram-negative				
*Escherichia coli* ATCC 0039	256	128	256	128
*Klebsiella pneumoniae* ATCC 1705 KPC^+^	256	>256	>256	>256
*Klebsiella pneumoniae* ATCC 1706 KPC^−^	256	>256	>256	>256
*Pseudomonas aeruginosa* ATCC 0027	256	128	256	128
Gram-positive				
*Staphylococcus aureus* ATCC 0023	>256	256	>256	256
*Enterococcus faecalis* ATCC 0033	>256	>256	>256	>256

**Table 2:** Potential synergistic effects between (anoplin)_2_K and known antimicrobial agents.

Bacteria	Antibiotic	MIC of Antibiotic (mg/L)	MIC of (anoplin)_2_K (µM)	Σ ICIF	Interpretation
*Escherichia coli*	CTX	1	128	0.75	Additive
		2	128	1.00	Additive
		2	64	0.75	Additive
		2	32	0.625	Additive
	SUT	8	32	0.75	Additive
		4	32	0.50	Synergistic
		32	32	2.25	Antagonistic
*Klebsiella pneumoniae* (KPC^−^)	CTX	1	64	0.75	Additive
	SUT	0.06	128	0.98	Additive
*Pseudomonas aeruginosa*	SUT	8	128	2.00	Indifferent
*Staphylococcus aureus*	CLI	0.03	128	0.74	Additive
		0.06	64	0.73	Additive
	SUT	0.03	256	1.50	Indifferent

Note: The fractional inhibitory concentration index (FICI) was calculated using the formula: FICI = (MIC of the peptide in combination/MIC of the peptide alone) + (MIC of the antimicrobial in combination/MIC of the antimicrobial alone). Values were interpreted as follows: synergism (FICI ≤ 0.5), additive effect (0.5 < FICI ≤ 1), indifferent effect (1 < FICI ≤ 2), and antagonism (FICI > 2). All tests were performed in triplicate.

## Data Availability

The original contributions presented in this study are included in the article/Supplementary Material. Further inquiries can be directed to the corresponding author.

## Supplementary Materials

The following supporting information can be downloaded at: https://www.mdpi.com/article/10.3390/microorganisms14081681/s1, Figure S1: Chromatographic profile of the peptide Anoplin and its (anoplin)_2_K. (A) (Anoplin), (B) (anoplin)_2_K; Figure S2: Mass spectrum of the peptide Anoplin and its (anoplin)_2_K. (A) (Anoplin), (B) (anoplin)_2_K.

## Abbreviations

MDR	Multidrug-resistant
AMRJ	Antimicrobial resistance
AMPs	Antimicrobial peptides
MIC	Minimum inhibitory concentration
MBC	Minimum bactericidal concentration
CFU/mL	Colony-forming units per milliliter
SPPS	Solid-phase peptide synthesis
Fmoc	9-fluorenylmethyloxycarbonyl
DMF	Dimethylformamide
DIC	Diisopropylcarbodiimide
HOBt	N-hydroxybenzotriazole
DCM	Dichloromethane (methylene chloride)
TFA	Trifluoroacetic acid
TIS	Triisopropylsilane
HPLC	High-performance liquid chromatography
UV	Ultraviolet
ESI	Electrospray ionization
LBM	Laboratory of Bacteriology and Mycology
UFJ	Federal University of Jataí
ATCC	American Type Culture Collection
BHI	Brain Heart Infusion
MHA	Mueller–Hinton agar
MHB	Mueller–Hinton broth
KPC	*Klebsiella pneumoniae* carbapenemase
μM	Micromolar
*v*/*v*	Volume/volume
HC_50_	Hemolytic concentration 50%
PBS	Phosphate-buffered saline
FICI	Fractional inhibitory concentration index
LPS	Lipopolysaccharide
