# Supplementary material for: Impact of Lysine-Mediated C-Terminal Dimerization of Anoplin on Killing Kinetics and Antibiotic Interaction Profiles
Source: Microorganisms. 2026 Jul 31;14(8):1681. doi: 10.3390/microorganisms14081681 (PMC13515977; doi:10.3390/microorganisms14081681)
Supplement: Supplementary file 1 [file microorganisms-14-01681-s001.zip › microorganisms-4462654-supplementary.pdf]

Supplementary material

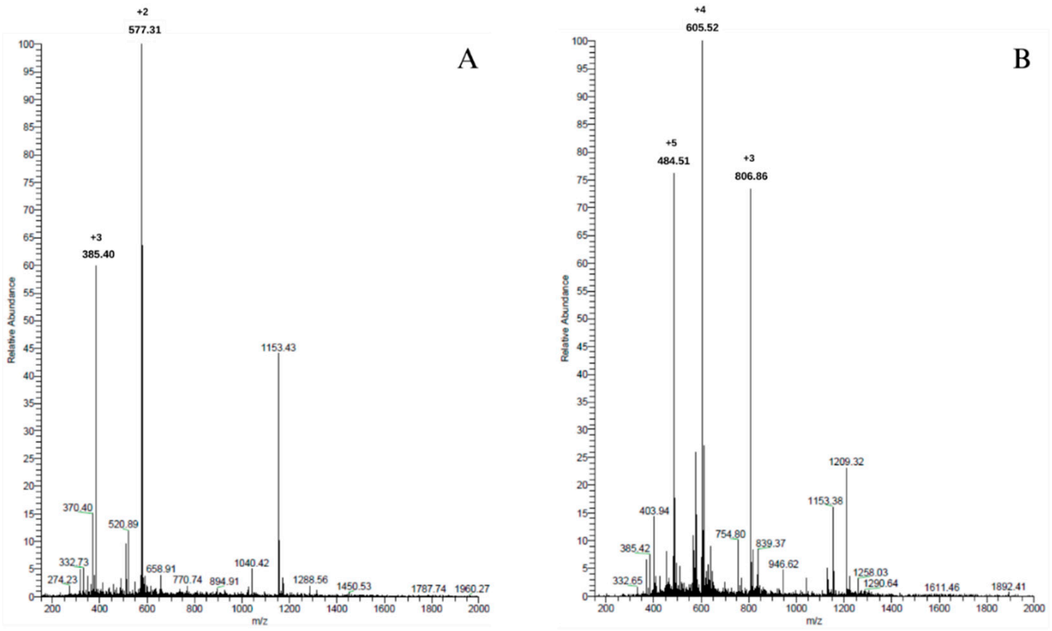

Figure S1. Chromatographic profile of the peptide Anoplin and its (anoplin)$_2$K. A - (Anoplin), B - (anoplin)$_2$K.

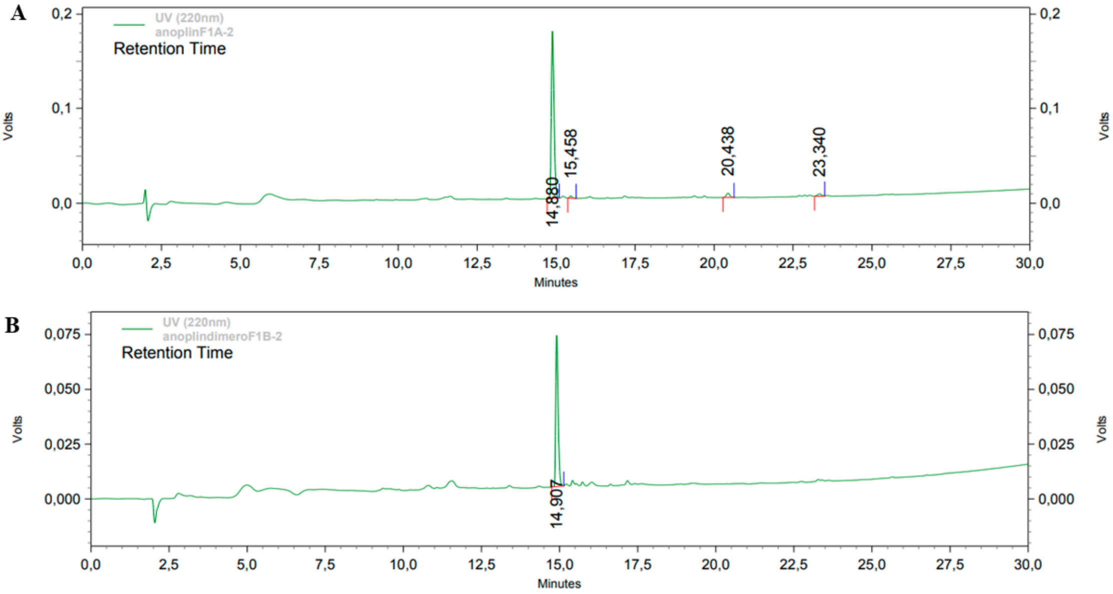

Figure S2. Mass spectrum of the peptide Anoplin and its (anoplin)$_2$K. A - (Anoplin), B - (anoplin)$_2$K.
